# Effect of Graphene Oxide on the Microstructural Properties and Self-Healing Capacity of Well Cement Slurries Cured for Nine Years

**DOI:** 10.3390/ma19214543

**Published:** 2026-10-28

**Authors:** Aleksandra Jamrozik, Grzegorz Malata, Rafał Wiśniowski, Karolina Jamrozik

**Affiliations:** 1 Faculty of Drilling, Oil and Gas, AGH University of Krakow, 30-059 Krakow, Poland; wisniows@agh.edu.pl; 2 Faculty of Materials Science and Ceramics, AGH University of Krakow, 30-059 Krakow, Poland; gmalata@agh.edu.pl; 3 Faculty of Medicine, University of Warmia and Mazury in Olsztyn, 10-162 Olsztyn, Poland; jamrozik.a.karolina@gmail.com

**Keywords:** graphene oxide, nanomaterials, well cement slurries, microstructural properties

## Abstract

The primary objective of well cementing is to achieve durable zonal isolation, thereby ensuring well integrity and safe long-term operation, which to a large extent depends on the proper design of the cement slurry formulation. In this context, particular importance is placed on the development of cement slurry formulations capable of withstanding harsh downhole conditions and producing set cement with modified microstructural properties and self-healing capacity. Graphene oxide (GO) is one of the potential nanoadditives that may support these mechanisms. Therefore, the aim of this study was to evaluate the long-term effect of GO on the microstructure and autogenous self-healing of microcracks in set well cement intended for wellbore sealing. Cement specimens prepared using CEM I 42.5 R Portland cement, with GO contents of 0.05% and 0.15% by weight of cement (bwoc), were examined after nine years of curing. Scanning electron microscopy (SEM) and micro-X-ray fluorescence (µ-XRF) analyses revealed microstructural features and elemental distribution patterns consistent with GO-induced modifications of the cement matrix. Observations of mineral precipitation within microcracks provided evidence of processes potentially contributing to autogenous crack sealing. Furthermore, porosity measurements demonstrated that the incorporation of GO into cement slurries with a water-to-cement ratio (w/c) of 0.7 reduces the pore volume, including the volume of capillary pores. These findings provide insights into the long-term microstructural behavior of GO-modified set cement and suggest its potential for developing advanced cement slurry formulations intended to enhance the durability of cement sheaths in hydrocarbon production, geothermal, and other demanding wellbore applications.

## 1. Introduction

Cementing of casing strings, including production casing strings, is one of the most important technological operations in well construction, both for wells drilled to access oil, natural gas, and geothermal water resources, and for injection wells used, among other purposes, for hydrogen storage or geological CO_2_ sequestration. The more primary objective of well cementing is to provide an effective seal in the annulus between the wellbore wall and the casing string. Proper wellbore sealing ensures durable zonal isolation of productive intervals, isolation of formation waters, prevention of formation-fluid leakage, and finally, mechanical support and corrosion protection of the casing strings, which perform specific functions once installed in the well [1,2].

The effectiveness of a well cementing process depends not only on the proper design and execution of the cementing job but also on the quality and properties of the cement slurry used. Portland cements or specialized Portland cements covered by API Specification 10A, commonly referred to as well cements, are primarily used in these operations and are modified with various additives and admixtures [3,4].

A properly designed cement slurry formulation should exhibit appropriate rheological and performance properties to ensure pumpability, facilitate effective displacement of drilling fluid, and achieve uniform cement slurry placement throughout the interval to be cemented. After setting and hardening, the set cement should contribute to maintaining well integrity and should exhibit low permeability to formation fluids as well as resistance to influence of corrosive formation fluids. It should be emphasized that cement sheath integrity in wellbores depends not only on the factors mentioned above but may also be affected by changes in pressure and temperature as well as by tectonic stresses [5]. Conventional set cements (without plasticizing admixtures) are quasi-brittle materials and exhibit low resistance to mechanical damage. Moreover, they lack the ability to autonomously heal microcracks.

Alberdi-Pagola and Fischer conducted research focusing primarily on the damage mechanisms of cement barriers [6]. Their results showed that an intact cement barrier in a wellbore is extremely rare. Accordingly, the use of additives and admixtures is essential to obtain cement slurry formulations with the required technological and microstructural properties as well as self-healing capacity, especially from a long-term perspective. Currently, nanomaterials have attracted widespread attention among additives used in cement slurry formulations [7]. The use of nanomaterials, particularly graphene oxide (GO), has emerged as a promising strategy for enhancing strength [8], improving microstructural properties [9], promoting self-healing [10], and increasing resistance to environmental factors [11].

The oxygen-containing functional groups of GO (e.g., –COOH, –OH, and epoxy groups) interact with Ca^2+^ ions and cement hydration products, acting as nucleation sites for the growth of calcium silicate hydrate (C–S–H), promoting the redistribution of portlandite, and reducing capillary porosity. These interactions accelerate cement hydration, densify the microstructure of the cement matrix, and strengthen interfacial bonding, resulting in improved compressive and flexural strength of set cement. The specific chemical structure of the surface functional groups of GO determines the strength and nature of these interactions, with edge –COOH groups forming particularly strong bonds with Ca^2+^ ions [12,13,14,15]. It should be emphasized that the benefits associated with the use of GO depend on its concentration in the cement matrix. Low dosages, typically 0.02–0.15% (bwoc), provide effective improvements in mechanical and microstructural properties [6]. However, higher GO concentrations lead to agglomeration and the formation of a heterogeneous microstructure, increased porosity, and reduced strength of set cement [16,17,18,19].

Previous studies investigating the effect of GO on the technological properties of cement slurries have yielded promising results. However, they have focused primarily on short-term changes, with observation periods of up to 365 days [11], or on accelerated aging scenarios. Whereas, the long-term effects of GO—particularly after nine years of aging—on the microstructural properties and self-healing capacity of set cement remain insufficiently investigated. Addressing this knowledge gap is of key importance for improving well integrity and the effectiveness of well cementing operations [20,21,22].

The novelty of this study lies in evaluating the microstructural evolution and self-healing capacity of GO-modified set cement over a nine-year period. The primary objective was to evaluate the influence of GO on the long-term microstructural properties of set cement. This work contributes to research aimed at developing advanced cement slurry formulations that may be used for cementing, for example, oil wells and geothermal [23] wells with the aim of maintaining long-term well integrity.

## 2. Materials and Methods

### 2.1. Materials

The materials used for the preparation of cement slurries included CEM I 42.5 R Portland cement (Górażdże Cement S.A., Chorula, Poland) and tap water supplied by Kraków Water (Kraków, Poland). Selected physicochemical properties of the CEM I 42.5 R Portland cement used in the study are presented in Table 1.

An aqueous graphene oxide dispersion was used as an additive in selected cement slurry formulations. GO purchased from Graphene Supermarket Inc. (Calverton, NY, USA). GO was obtained by oxidation of graphite using a mixture of sulfuric acid, sodium nitrate, and potassium permanganate according to the Hummers method. The dark-brown aqueous GO dispersion had a graphene oxide concentration of 6.2 g/dm^3^ The elemental composition included 79% carbon and 20% oxygen. The proportion of single-layer flakes exceeded 80%. The thickness of an individual GO flake was 1 nm, while its lateral size ranged from 0.5 to 5.0 μm. The BET specific surface area of GO was 833 m^2^/g.

### 2.2. Methods

The study involved experimental investigations into the effect of an aqueous dispersion of graphene oxide in the form of large-area flakes on microstructural modification and restructuring, the associated transport properties, and the mechanisms responsible for maintaining the self-healing capacity of set cement after a long-term, nine-year curing period (specimens designated N0.7, N0.7 + 0.05% GO, and N0.7 + 0.15% GO). These effects were compared with those observed in set cement after 28 days of hydration (specimens designated S0.7, S0.7 + 0.05% GO, and S0.7 + 0.15% GO).

The cement slurries were prepared in accordance with PN-EN ISO 10426-2 [25], using the materials described in Section 2.1. The water-to-cement ratio (w/c) was 0.7, and the slurries were modified with GO additions of 0%, 0.05% and 0.15% (bwoc). A water-to-cement ratio of 0.7 was selected because cement pastes prepared at this ratio exhibit high porosity and permeability after hardening [26,27], which may render them more susceptible to carbonation. The aqueous GO dispersion was first mixed into the mixing water, after which cement was added in accordance with the procedure specified in PN-EN ISO 10426-2 [25]. The water introduced with the GO dispersion was included in the total mixing-water content when calculating the water-to-cement ratio.

Cylindrical specimens with a diameter of 38 mm and a height of 80 mm were prepared. All specimens were prepared during the same experimental series, using the same raw materials and the specified mixture proportions for each formulation, and were subjected to identical initial curing conditions. During the initial 28-day hydration period, the specimens were cured in a water bath maintained at 23 ± 2 °C. Subsequently, the specimens designated for long-term curing remained in the same water bath at 23 ± 2 °C until the 90th day. After this period, the specimens were subjected to alternating curing cycles consisting of 90 days of exposure to an environment with a relative humidity of 55–65% at 23 ± 2 °C, followed by 90 days of immersion in a water bath maintained at the same temperature. These alternating cycles were continued until the specimens reached a total age of nine years.

The specimens subjected to long-term curing originated from the same experimental series as those examined after 28 days of hydration. Thus, the nine-year study represents a continuation of the original experimental program, ensuring consistency in material composition, specimen preparation, and initial curing conditions.

Porosimetric measurements were performed on set cement specimens after their respective curing periods to determine the specific surface area, pore-size distribution, and pore volume. Separate specimens from the same experimental series, cured for either 28 days or nine years, were used for further characterization of their elemental distribution, microstructure, and phase composition using micro-X-ray fluorescence (µ-XRF) Bruker, Billerica, MA, USA, scanning electron microscopy (SEM) NOVA NANO SEM 200, Eindhoven, The Netherlands, and X-ray diffraction (XRD) PHILIPS PW-1040, Eindhoven, The Netherlands.

#### 2.2.1. Micro-X-Ray Fluorescence Spectrometry (μ-XRF)

μ-XRF analysis was used to generate elemental distribution maps of selected elements in cross-sections cut from the cores of nine-year-old set cement specimens. For this purpose, set cement specimens cured for 28 days and for nine years were cut to expose their internal cross-sections. The freshly exposed surfaces were cleaned and polished prior to analysis with an M4 Tornado Plus scanning X-ray fluorescence microanalyzer (Bruker, Billerica, MA, USA).

Measurements were performed using an X-ray tube with an Rh anode operated at 50 kV and 600 μA. The excitation beam was focused using a polycapillary lens, and the pixel size was approximately 25 μm. Measurements were carried out in surface-scanning mode on polished sections approximately 0.9 mm thick, cut from the cores of the set cement specimens. The dwell time per pixel was 40 ms. The specimens were mapped continuously at a sample-stage speed of 500 μm/s under low-vacuum conditions (2 mbar). The characteristic X-ray radiation of the elements was recorded using two silicon drift detectors (SDDs).

Data analysis was performed using the dedicated M4 Tornado Plus software ESPRIT 1.5. Based on the intensities of the characteristic Kα X-ray lines on the elements at individual measurement points, elemental distribution maps were generated and compared with the corresponding surface images of the analyzed specimens.

#### 2.2.2. Visual Assessment

The freshly exposed cross-sectional surfaces of nine-year-old set cement specimens were subjected to optical observation using an Evident SZX10 stereomicroscope at 15× magnification (Evident, Needham, MA, USA). After image documentation had been completed, the specimen surfaces were cleaned of loose particles, and the “RAINBOW Indicator” (Germann Instruments, Copenhagen, Denmark) was then uniformly applied by spraying. After the indicator had completely dried, photographic documentation of the resulting coloration was obtained and compared with the manufacturer’s reference color scale. On this basis, a semi-quantitative assessment of the surface pH was performed using the following ranges: 4–6—orange, 6–8—yellow, 8–10—green, 10–12—violet, and 12–14—blue. The assessment was performed without magnification, under identical lighting conditions, with a constant camera-to-specimen distance and identical image acquisition settings.

The analysis included the coloration of the main cement matrix, near-surface zones, cracked areas, and products forming secondary infill of the cracks. Particular attention was paid to local differences in coloration between the material filling the cracks and the surrounding cement matrix. The indicator test was used exclusively for the semi-quantitative assessment of the spatial variation in surface pH. Crystalline mineral phases were identified by X-ray Diffraction (XRD), whereas the microstructure and local elemental composition were characterized by scanning electron microscopy (SEM-EDS) NOVA NANO SEM 200 (Eindhoven, The Netherlands).

#### 2.2.3. X-Ray Diffraction

Samples for phase composition analysis by X-ray diffraction were collected from the cores of set cement specimens after 28 days and nine years of hydration. To determine the phase distribution as a function of depth, samples were collected from successive 0.25 mm-thick layers over a depth range of 0–3 mm from the surface toward the interior of the specimen core. After drying to constant mass, the samples were ground in an agate mortar until the entire material passed through a 0.063 mm sieve.

The prepared samples were analyzed using the Debye–Scherrer–Hull powder diffraction method with a PHILIPS PW-1040 X-ray diffractometer (Eindhoven, The Netherlands). The measurement parameters were as follows: current of 16 mA, voltage of 35 kV, step size of 0.05°, and counting time of 3 s per step. The angular measurement range was 5–65° 2θ, whereas the diffractograms presented in the figures were limited to the range of 10–45° 2θ. Phase identification was performed using the PDF-2 database and X’Pert HighScore Plus 5.3a software.

#### 2.2.4. Scanning Electron Microscopy (SEM) with Energy-Dispersive X-Ray Spectroscopy (EDS)

The microstructure of fracture surfaces of 28-day-old and nine-year-old set cement specimens modified with graphene oxide was investigated using an Nova NanoSEM 200 scanning electron microscope (Eindhoven, The Netherlands) equipped with a field-emission gun (FEG, Schottky emitter) and coupled with an EDAX energy-dispersive X-ray spectroscopy (EDS) system for elemental analysis. The spatial resolution of the microscope was 2 nm, and the magnification range was 70–500,000×. Observations were conducted under low-vacuum conditions at a chamber pressure of 60 Pa. Prior to examination, the unpolished fracture surfaces of the specimens were coated with a thin layer of carbon to prevent the accumulation of electrostatic charge during observation.

#### 2.2.5. Porosimetric Analysis

The specific surface area, pore-size distribution, and pore volume were determined by nitrogen (N_2_) sorption using an ASAP 2020 HD analyzer (Micromeritics Inc., Norcross, GA, USA) and by mercury (Hg) intrusion porosimetry using a PoreMaster 60 mercury porosimeter (Anton Paar, Graz, Austria). The measurements were performed on crushed specimens that were degassed at 120 °C for 24 h prior to analysis.

## 3. Results and Discussion

### 3.1. Elemental Mapping by Micro-X-Ray Fluorescence Spectrometry (μ-XRF)

**Figure 1:**
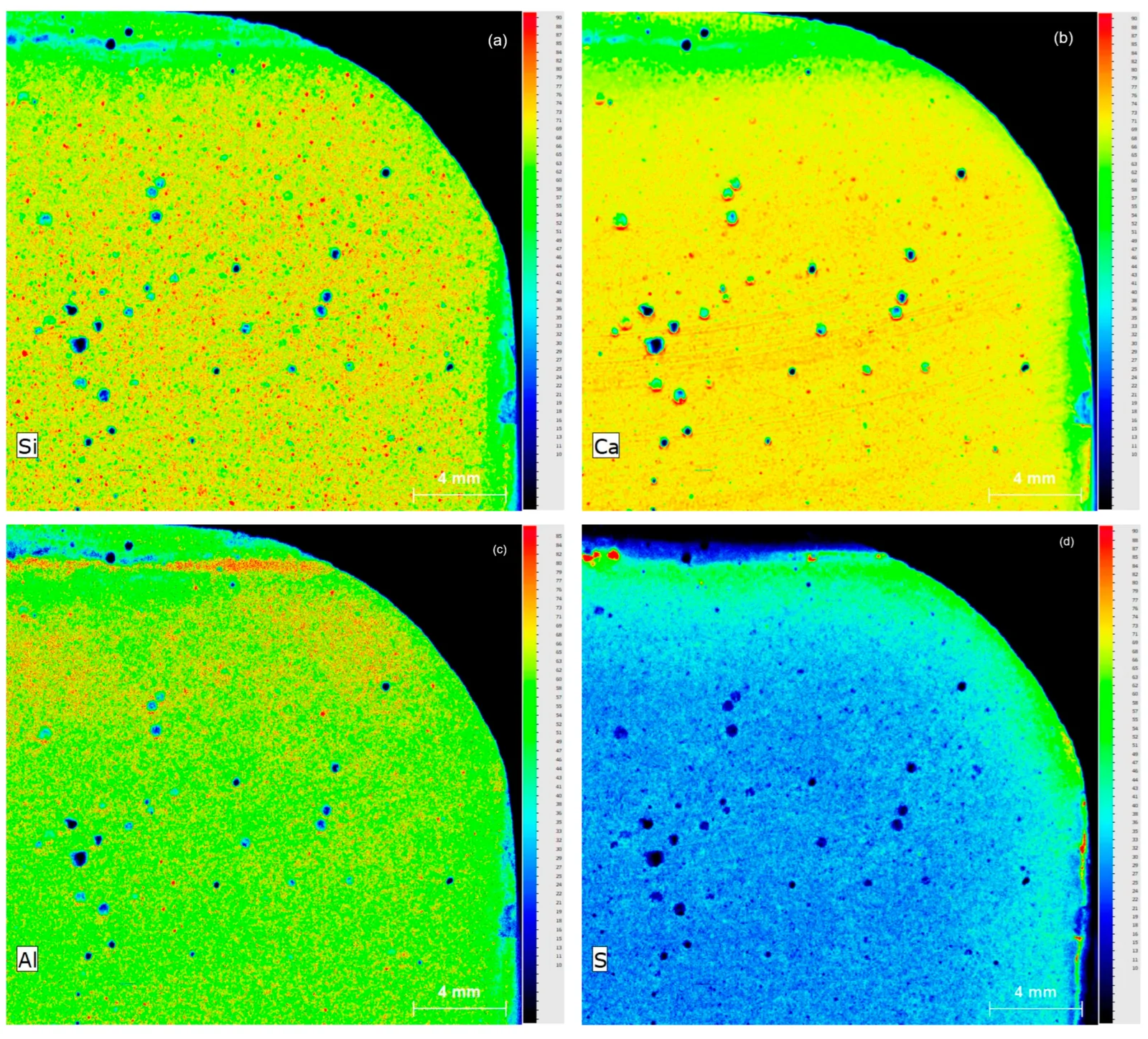
Elemental distribution maps on the polished surfaces of nine-year-old set cement specimens without GO, obtained by μ-XRF: (**a**) silicon (Si) distribution map; (**b**) calcium (Ca) distribution map; (**c**) aluminum (Al) distribution map; (**d**) sulfur (S) distribution map.

**Figure 2:**
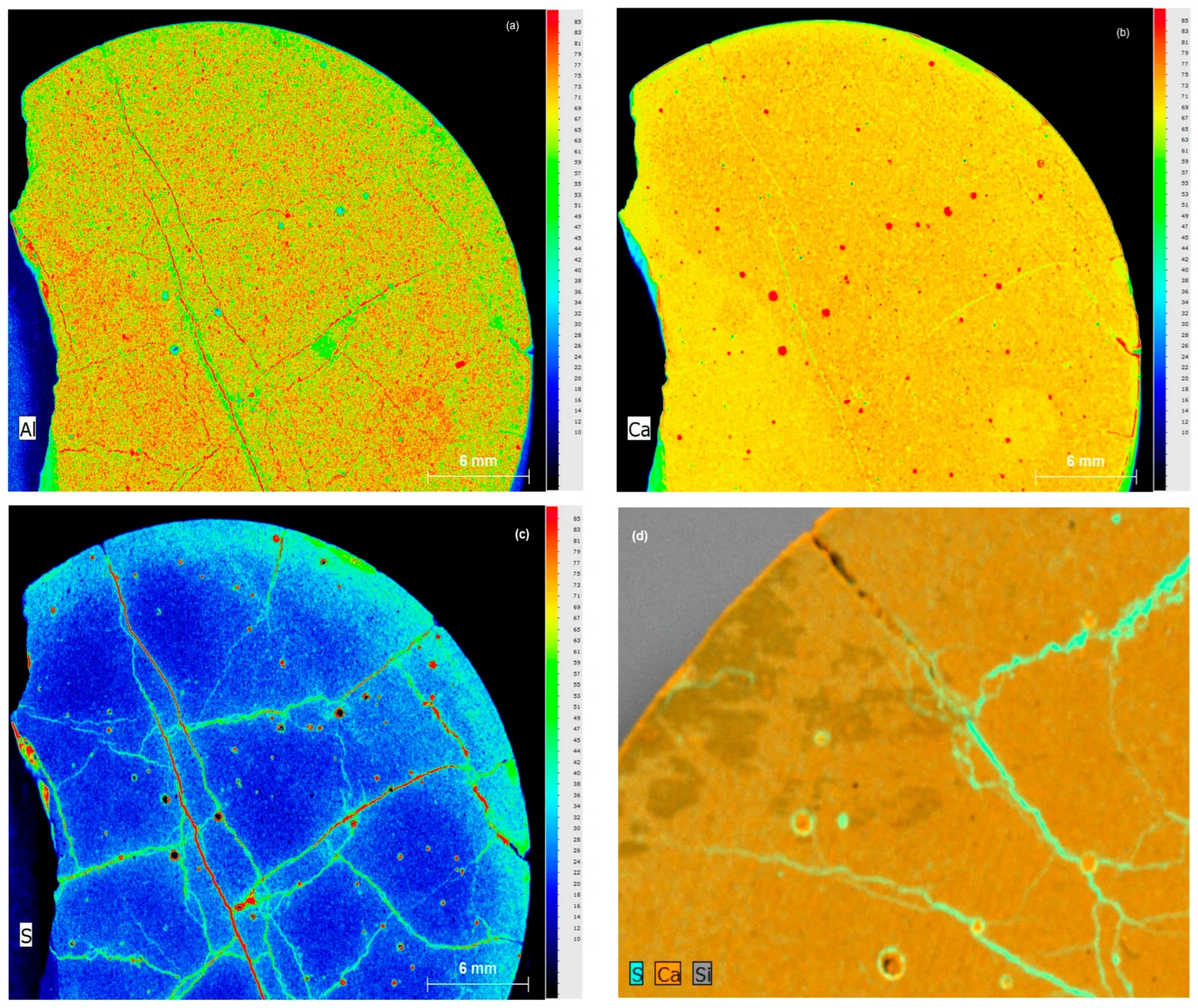
Elemental distribution maps on the polished surfaces of nine-year-old set cement specimens containing 0.05% GO (bwoc), obtained by μ-XRF: (**a**) aluminum (Al) distribution map; (**b**) calcium (Ca) distribution map; (**c**) sulfur (S) distribution map; (**d**) composite μ-XRF distribution map of sulfur (S), calcium (Ca), and silicon (Si).

**Figure 3:**
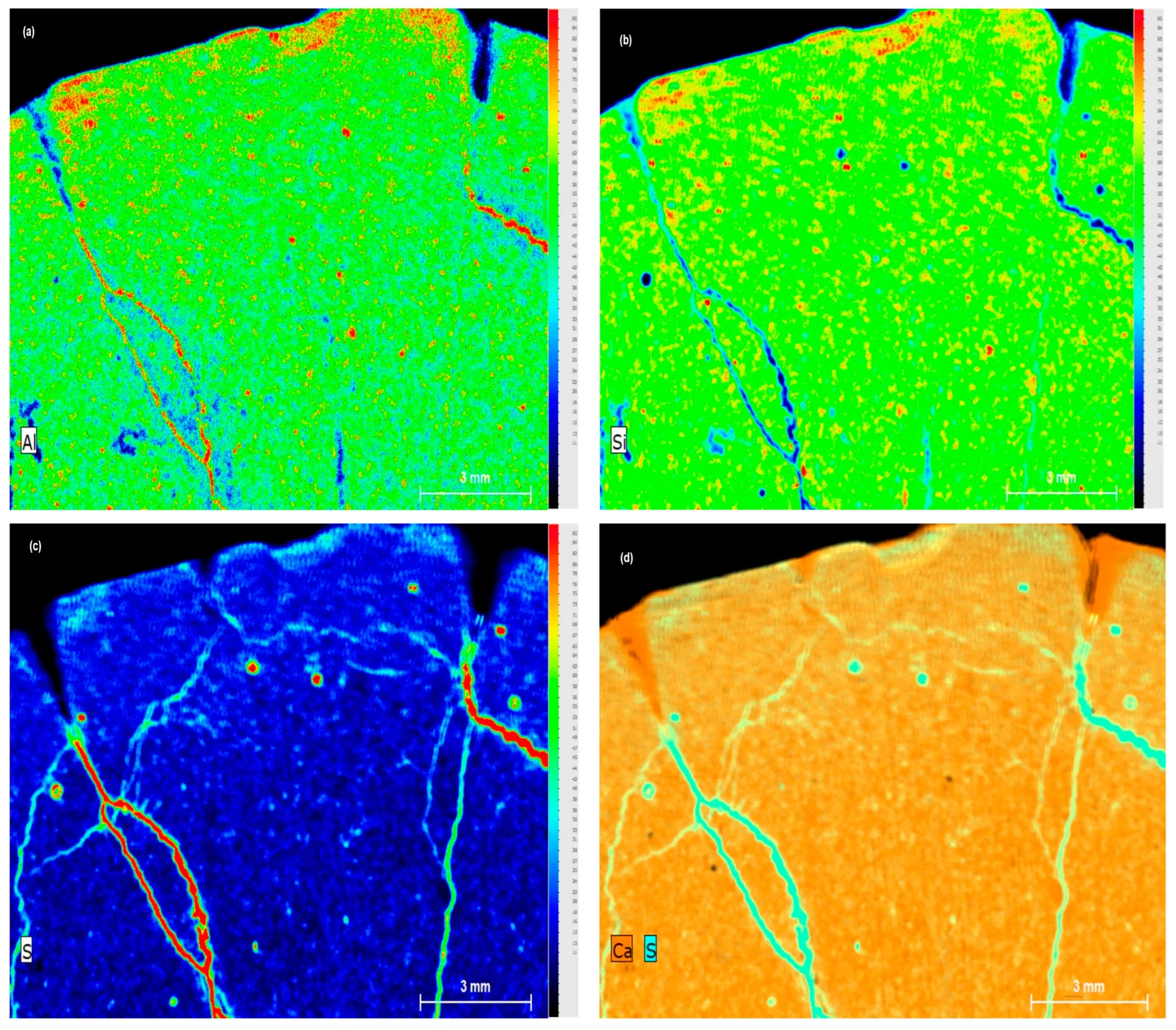
Elemental distribution maps on the polished surfaces of nine-year-old set cement specimens containing 0.15% GO (bwoc), obtained by μ-XRF: (**a**) aluminum (Al) distribution map; (**b**) silicon (Si) distribution map; (**c**) sulfur (S) distribution map; (**d**) composite μ-XRF distribution map of calcium (Ca) and sulfur (S).

The distribution of selected elements was analyzed in polished thin cross-sections prepared from the cores of nine-year-old set cement specimens, including reference specimens without GO and specimens containing 0.05% and 0.15% GO by weight of cement (bwoc). The analysis was performed using an M4 TORNADO PLUS micro-X-ray fluorescence (µ-XRF) spectrometer (Bruker, Billerica, MA, USA). The resulting elemental distribution maps are presented in Figure 1, Figure 2 and Figure 3.

Analysis of the elemental of distribution in nine-year-old set cement specimens without GO (Figure 1) revealed relatively uniform distributions the main elements (Si, Ca, Al, and S), except for localized elemental depletion in pore regions and sulfur enrichment in near-surface deposits.

Elemental mapping of GO-containing set cement specimens (Figure 2 and Figure 3) revealed two distinct patterns of void filling within the cement matrix. In the first case, air voids, originating during cement slurry preparation exhibited evidence of mineral deposition, potentially resulting from the migration and precipitation of dissolved species. Calcium enrichment was observed throughout the pore cross-sections, whereas sulfur enrichment was concentrated near the pore walls, accompanied by markedly reduced aluminum and silicon signals. This elemental distribution suggests the possible precipitation of calcium sulfate phases, potentially including gypsum, along the pore walls and the subsequent accumulation of calcium-rich phases, such as portlandite, within the pores.

Cracks which provide pathways for rapid diffusion through the set cement are filled in their central parts with material enriched in aluminum and sulfur, accompanied by a relative decrease in calcium content. In a limited number of areas where an increase in sulfur content is not accompanied by a corresponding increase in aluminum content, the presence of gypsum-bearing material may be expected. Crack spaces located near the surface of the set cement—and locally the surface itself—are enriched mainly in calcium-concentrating material.

Another interesting observation is that the filling of free spaces with calcium and sulfur compounds did not affect the distribution of these elements within the specimen volume. In contrast, aluminum, which has a lower diffusion potential, migrated into the open crack space exclusively from the immediate surroundings, leaving aluminum-deficient areas parallel to the filled cracks.

### 3.2. Visual Observations

**Figure 4:**
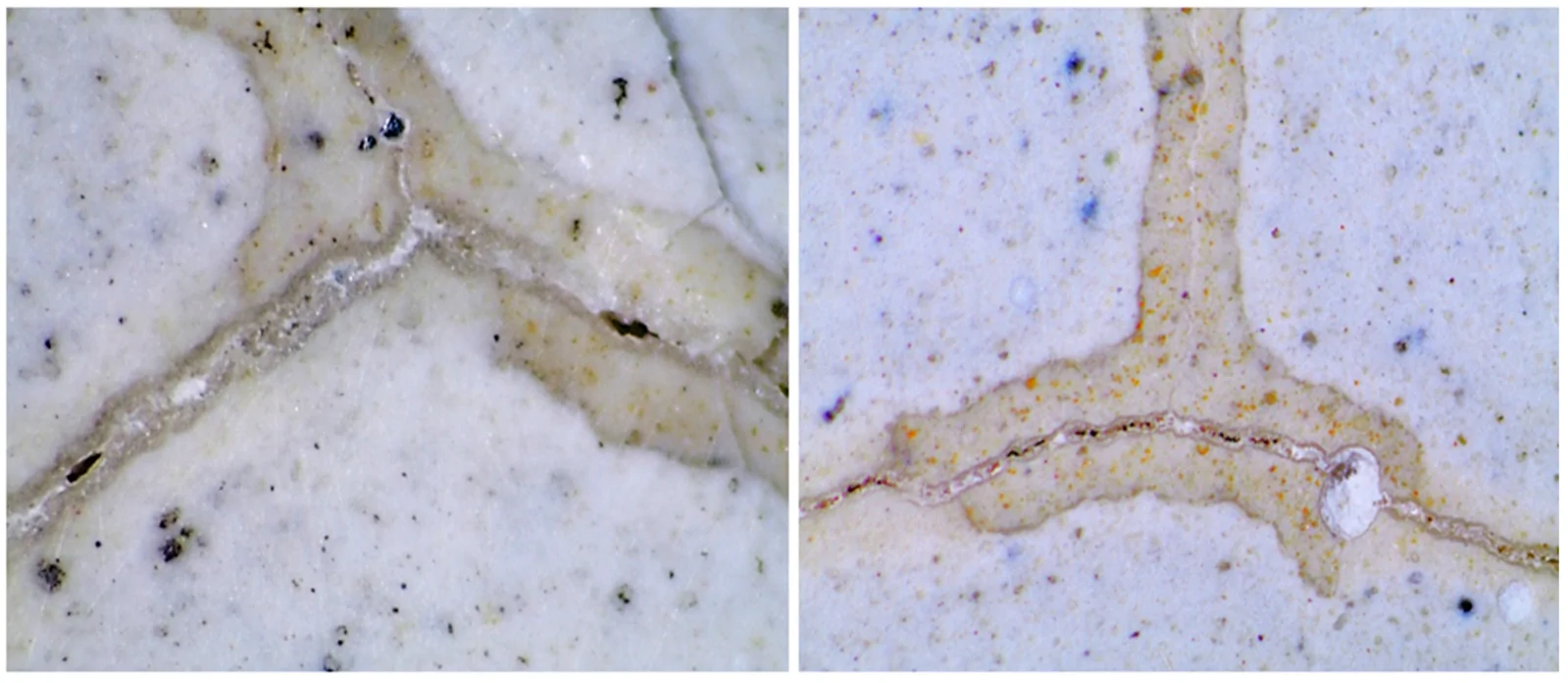
Macrographs of microcracks being infilled with a white crystalline product in nine-year-old set cement containing 0.15% GO (bwoc), acquired using an Olympus Evident SZX10 stereomicroscope at 15× magnification.

**Figure 5:**
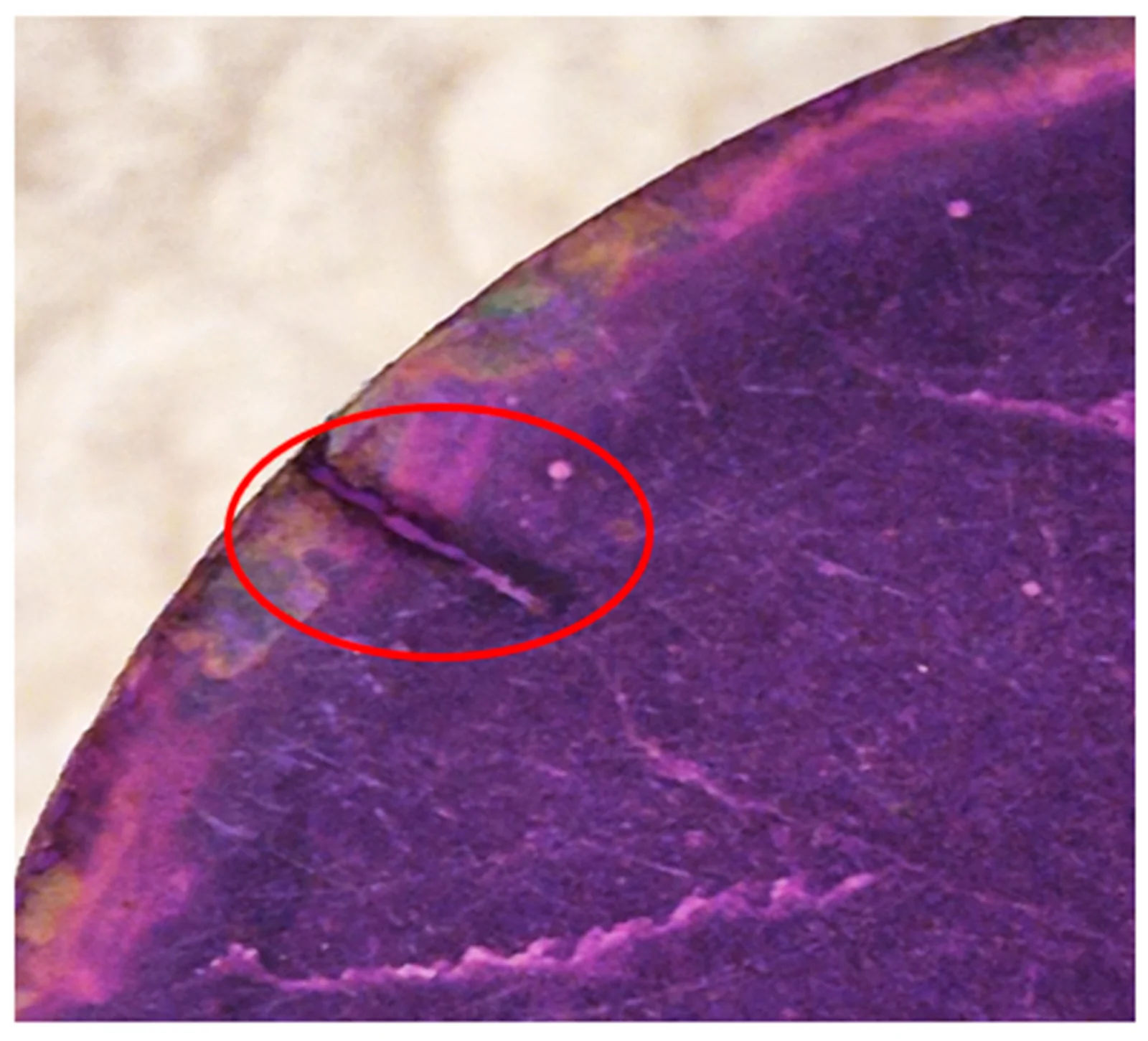
Macrograph of a nine-year-old set cement containing 0.15% GO (bwoc). Specimen stained with the Rainbow Test indicator, showing cracks healed with calcium hydroxide (portlandite) and a thin neutralized near-surface zone containing calcium carbonate (calcite). The red circle indicates the area of the filled microcrack.

In light of the results of the elemental distribution analysis (Figure 1, Figure 2 and Figure 3), macroscopic observations of microcracks were carried out on the freshly exposed surface of nine-year-old set cement (Figure 4), both before and after staining the surface with the Rainbow Test pH indicator solution (Figure 5).

The obtained results indicate that calcium hydroxide may be present or may form within the specimen and within the youngest, secondarily infilled microcracks. Meanwhile, calcium carbonate is more likely to occur on the specimen surface and at the outlets of older microcracks.

### 3.3. X-Ray Diffraction (XRD)

**Figure 6:**
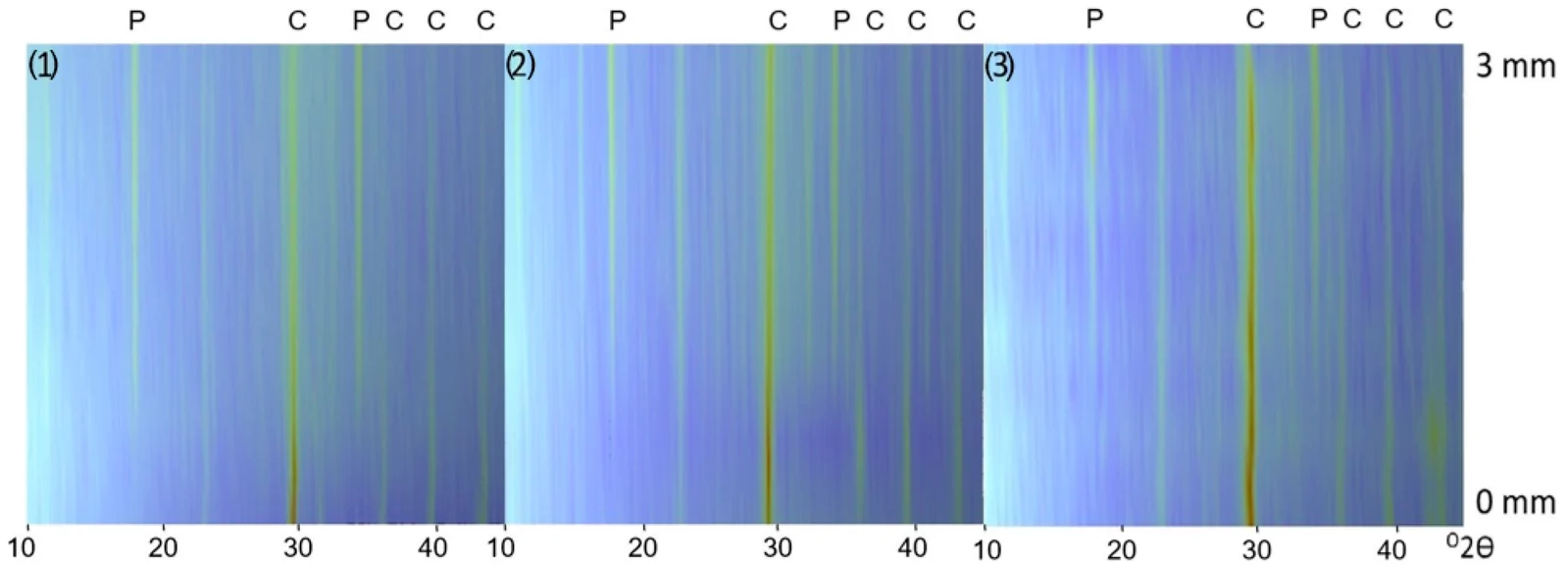
X-ray diffractograms presented as contour plots versus depth (from 0 to 3 mm) for nine-year-old set cement specimens containing: (**1**) 0.15% GO (bwoc), (**2**) 0.05% GO (bwoc), (**3**) without GO; P—portlandite, C—calcite. The depth origin (0 mm) corresponds to the original external specimen surface after removal of secondary surface crystallization products. Depth is measured inward from this surface.

In the context of the previously identified microstructural restructuring of the set cement, including, among other effects, the infilling of microcracks observed in specimens after nine years of curing and the presence of a neutralized surface layer on the set cement specimens (Figure 5), the next stage of the study involved an analysis of the phase composition of nine-year-old set cement specimens with a w/c ratio of 0.7, both with and without GO. The X-ray diffractograms are presented as contour plots as a function of measurement depth (0–3 mm) for nine-year-old set cement specimens containing 0.15% GO (bwoc), 0.05% GO (bwoc), and no GO (Figure 6).

Analysis of the X-ray diffractograms presented as a function of measurement depth revealed differences in phase composition between nine-year-old set cement specimens modified with graphene oxide and the reference set cement without GO (Figure 6). In the specimens containing 0.15% GO (bwoc) and 0.05% GO (bwoc), intense reflections assigned to calcite were observed predominantly in the near-surface layer, whereas portlandite dominated in the bulk of the specimens. This may indicate a limited depth of carbonation within the GO-modified set cement. A different pattern was observed in the set cement specimens without GO. In these samples, reflections assigned to calcite (C) were present throughout the entire analyzed depth (Figure 6(3)). This indicates deeper carbonation of this set cement.

**Figure 7:**
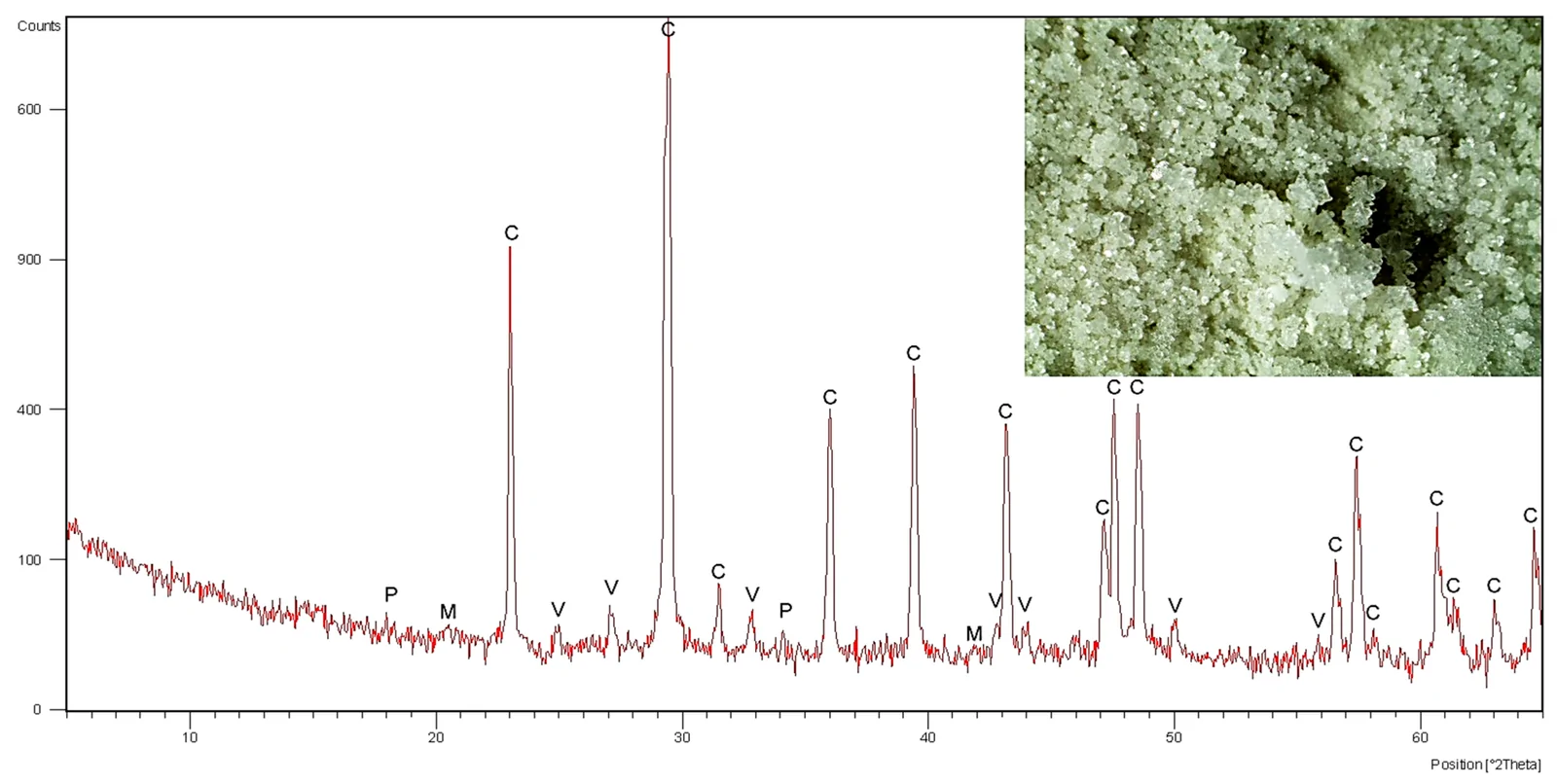
X-ray diffractogram of the products formed on the freshly exposed surface of nine-year-old set cement containing 0.15% GO (bwoc); P—portlandite, C—calcite, V—vaterite, M—monohydrocalcite.

The presence of portlandite in the deeper zones of the GO-modified specimens may be associated with the preservation of the alkaline character of the cement matrix, which is important for material durability and secondary sealing of the microstructure through the infilling of microcracks. Furthermore, the rapid formation of a white crystalline layer on the freshly exposed crack surface of the nine-year-old set cement during drying suggests the occurrence of secondary precipitation of crystalline phases within the crack. The resulting products were carefully collected using a sharp tool to obtain a sufficient amount of powder for X-ray diffraction (XRD) analysis. The diffractogram of the material collected from the crack surface is presented in Figure 7. The diffractogram reflects the overall phase composition of the collected products, consisting predominantly of a mixture of calcite (C) and vaterite (V), with possible contributions from portlandite (P) and monohydrocalcite (M).

### 3.4. Scanning Electron Microscopy (SEM) with EDS Analysis

**Figure 8:**
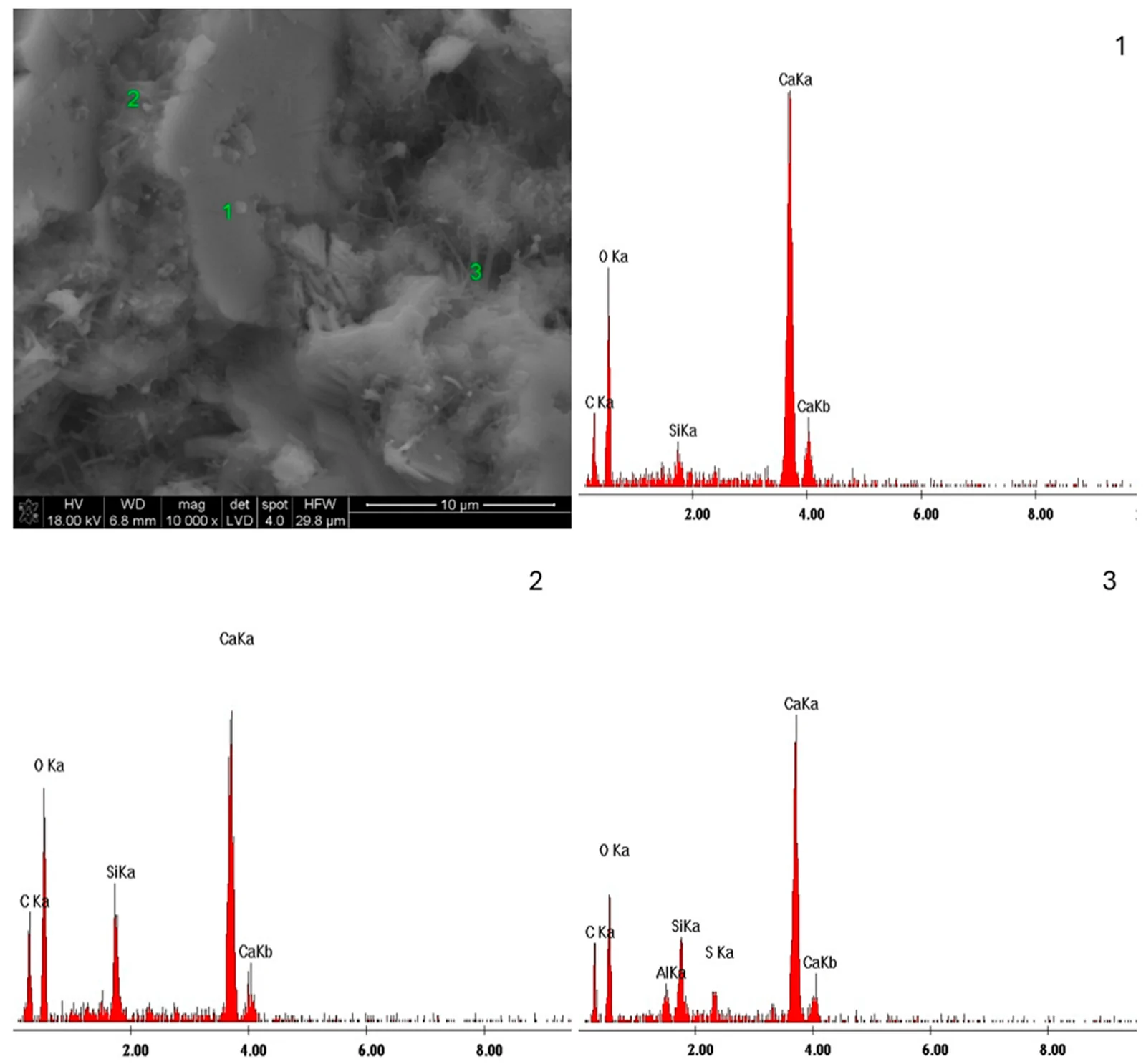
SEM micrograph of the set cement specimen without GO after 28 days of hydration, at 10,000× magnification. Elemental analysis of selected microareas at points (**1**–**3**) was performed using SEM–EDS.

**Figure 9:**
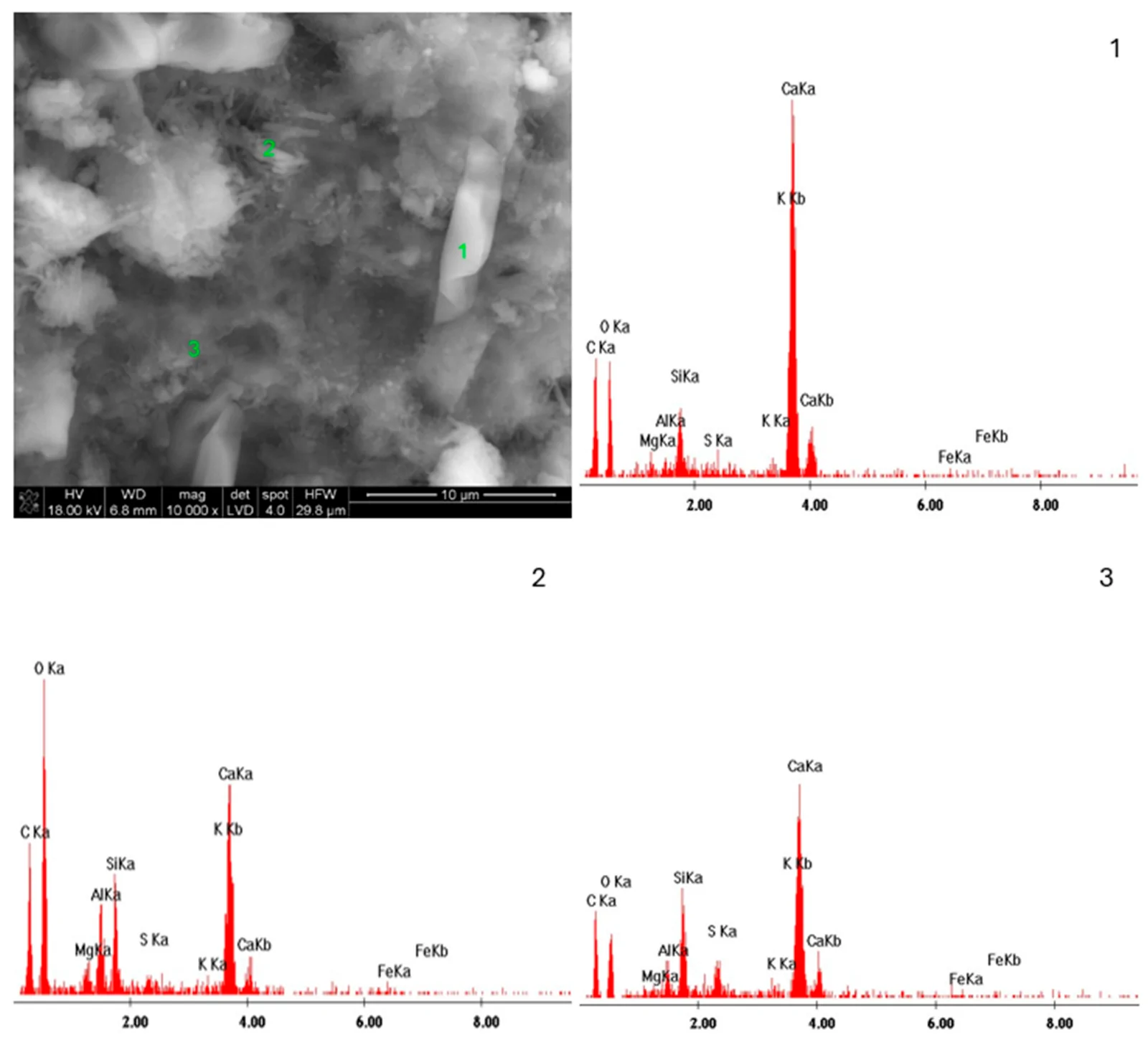
SEM micrograph of the set cement specimen containing 0.15% GO (bwoc) after 28 days of hydration, at 10,000× magnification. Elemental analysis of selected microareas at points (**1**–**3**) was performed using SEM–EDS.

**Figure 10:**
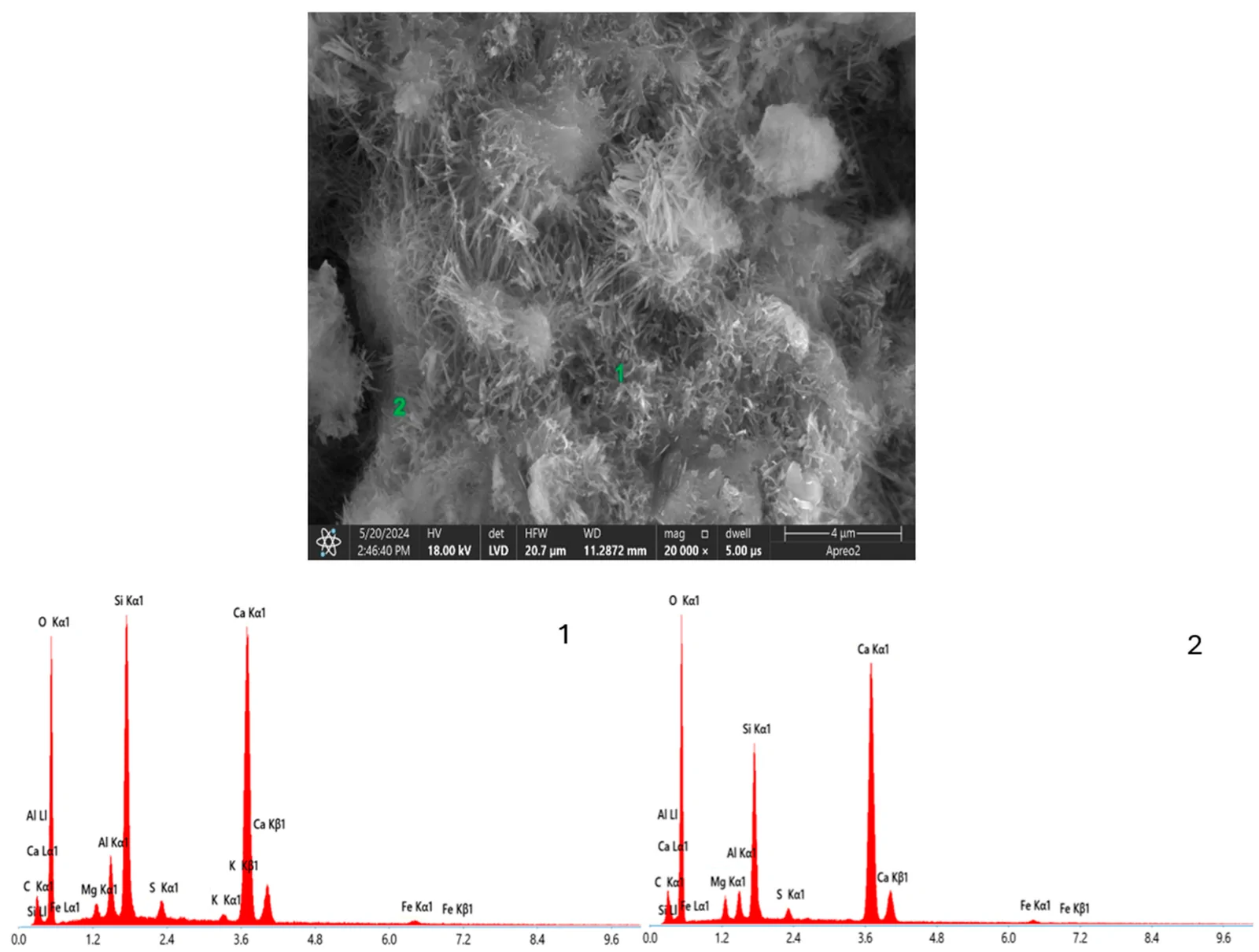
SEM micrograph of the set cement specimen without GO after nine years of hydration, at 20,000× magnification. Elemental analysis of selected microareas at points (**1**,**2**) was performed using SEM–EDS.

**Figure 11:**
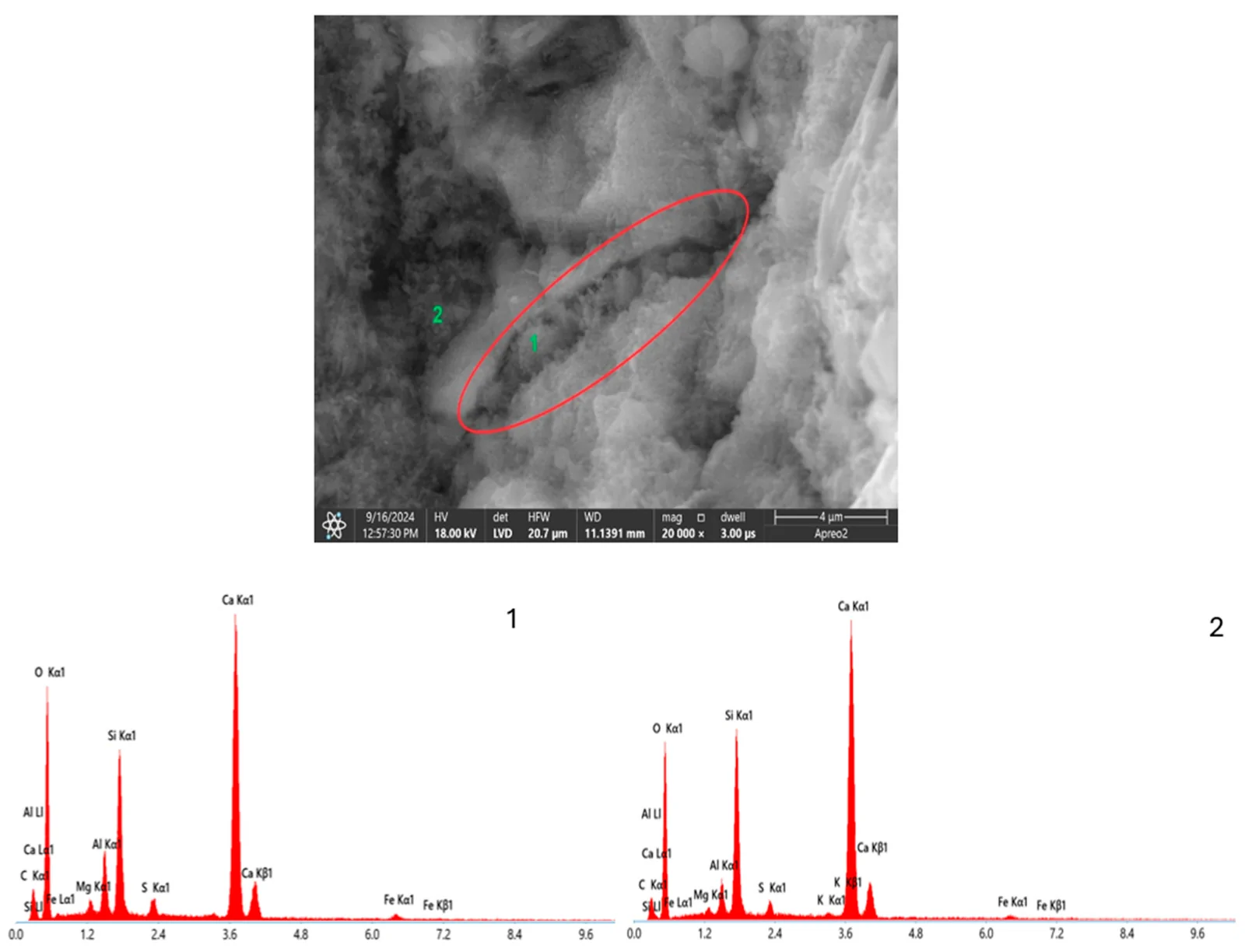
SEM micrograph of the set cement specimen containing 0.15% GO (bwoc) after nine years of hydration, at 20,000× magnification. Elemental analysis of selected microareas at points (**1**,**2**) was performed using SEM–EDS. The red circle indicates the area of the filled microcrack.

To further interpret the μ-XRF and XRD results and to relate the observed spatial variation in the phase composition of the specimens to the reorganization of the microstructure of the set cement specimens, observations were carried out using scanning electron microscopy with energy-dispersive X-ray spectroscopy (SEM–EDS). SEM–EDS analysis enables the identification of the morphology of hydration products, the degree of matrix densification, and the nature of secondary infilling of microcracks. SEM–EDS observations of the analyzed set cement specimens after 28 days and nine years of hydration are presented in Figure 8, Figure 9, Figure 10 and Figure 11.

Figure 8 shows the microstructure of the 28-day-old set cement without GO, whereas Figure 9 shows the microstructure of set cement containing GO. Typical hydration products of Portland cement can be observed in these figures, in particular portlandite (Figure 8 and Figure 9, point 1 of the EDS analysis), the C–S–H phase (Figure 8 and Figure 9, point 2), and the C–S–H phase covering ettringite crystals (Figure 8 and Figure 9, point 3 of the EDS analysis). The effect of graphene oxide is manifested by densification of the microstructure of the C–S–H phase (points 2 and 3 in Figure 9) and by changes in crystallite habit (e.g., portlandite in the form of a thin platelet—see point 1 in Figure 9).

Observations of the microstructure of the nine-year-old set cement specimens presented in Figure 10 and Figure 11 indicate that the incorporation of GO into the cement slurry formulation affects the restructuring of the main cement hydration products, which, in the absence of GO, predominantly exhibit a loosely packed, needle-like, well-crystallized morphology (see Figure 10, point 1).

The incorporation of GO results in substantial densification of the microstructure of the nine-year-old set cement specimens, which is composed of very fine, intergrown crystallites forming a felt-like microstructure (Figure 11). In addition, it was observed that the matrix composition differs between the areas occupied by the original hydration products and the products precipitated within the microcracks, where a distinct enrichment in aluminate-sulfate products (probably ettringite) occurs. Elemental composition analysis showed that the aluminum (Al) content detected at point 1 in Figure 11 was approximately twice that detected at point 2 in Figure 10.

The microstructural investigations presented in Figure 11 are consistent with observations reported by the other authors. In the study conducted by G. Li et al. (2019) [28], it was observed that graphene oxide influenced the formation of connections between the cement matrix and ettringite. Ettringite was reported to fill pores and cracks in the set cement. The filling of pores is manifested by the complete disappearance of capillary porosity measured by mercury intrusion porosimetry [27]. According to G. Li et al. (2019) [28], the C–S–H phase covers the graphene surface and immobilizes the contact point between graphene and ettringite. One end of the ettringite crystal was in contact with graphene, while the other was in contact with the cement matrix, thereby forming a bridge between the two components. This arrangement suggests the formation of a three-dimensional graphene–ettringite structure interacting with the surrounding cement matrix. The associated spatial variations in elemental distribution were also observed in the µ-XRF analyses presented in Figure 2 and Figure 3.

Research conducted by Wan et al. [29] indicates that the mechanism by which GO modifies the cement matrix changes over time. The authors analyzed these changes during the first 28 days of hydration, demonstrating that, in the early stages, well-dispersed GO primarily accelerates hydration. The results obtained in the present study after nine years of curing indicate that GO has a beneficial effect on microstructural densification and promotes microcrack filling and bridging in hardened cement paste, thereby supporting the material’s capacity to heal microcracks (see Figure 3, Figure 4 and Figure 11).

According to the authors, the formation of ettringite bridges within the microcracks promotes their gradual infilling and, consequently, contributes to a reduction in porosity and an improvement in the long-term durability of the investigated set cement specimens. However, the extrapolation of the results of microstructural densification and microcrack healing studies to mechanical properties requires further experimental verification.

### 3.5. Pore Structure Characterization

**Figure 12:**
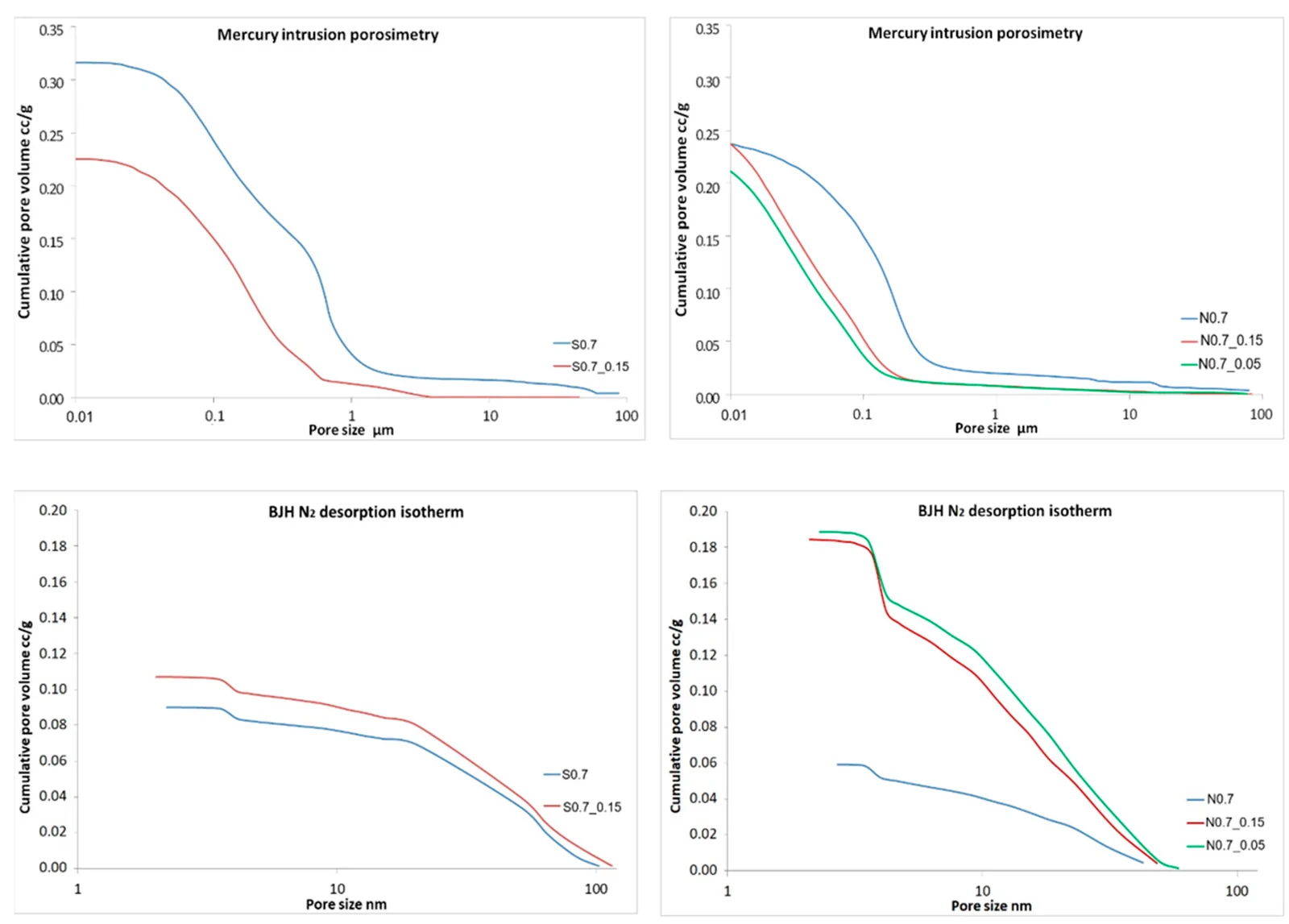
Comparison of porosimetric results obtained using Hg and N_2_ for set cement specimens after 28 days of hydration without GO (S0.7) and with 0.15% bwoc GO (S0.7_0.15), as well as for nine-year-old set cement specimens without GO (N0.7), with 0.05% bwoc GO (N0.7_0.05), and with 0.15% bwoc GO (N0.7_0.15).

In the next stage, porosimetric analyses using Hg and N_2_ were performed on set cement specimens after 28 days and after a nine-year curing period. These investigations were carried out based on the previously identified microstructural restructuring of the nine-year-old set cement specimens, including, among other effects, the infilling of microcracks. The results are presented in Table 2 and Figure 12.

Analysis of the selected porosity parameters presented in Table 2 and Figure 12 shows that the incorporation of graphene oxide into the cement slurry, already at an early stage of curing (28 days), was associated with a significant reduction in porosity, particularly with the elimination of a substantial proportion of fine capillary pores responsible for pore-fluid transport in the set cement. This reduction was accompanied by a slight increase in the proportion of finer pores, including gel pores. Over the extended curing period (nine years), a further reduction in capillary porosity occurred, together with a decrease in the upper limit of the capillary pore-size range in the set cement.

At the same time, the specimens containing graphene oxide exhibited a substantial, nearly more than three times increase in the proportion of finer pores. The lower limit of the pore-size range within which this increase occurred corresponded to the size of gel pores in the C–S–H phase [30,31]. This increase in porosity was accompanied by an approximately fourfold increase in specific surface area, as determined by N_2_ adsorption/desorption measurements.

Analysis of pore sizes using different calculation models showed that the restructuring of the pore-size distribution affected the mesopore range to a lesser extent, with an approximately more than three times decrease in the average pore size determined by the BJH method, whereas a much greater change was observed in the micropore range. Within the BET range, the average pore size decreased by a factor of approximately 20, reaching values below 1 nm.

The specific surface area of the nine-year-old set cement (approximately 19 m^2^/g) is consistent with values reported in the literature for mature set cement with the same water-to-cement ratio [32]. The increase in specific surface area observed in the graphene oxide-modified specimens may be associated with various effects. Referring to the long-recognized dependence of the resulting specific surface area on the humidity conditions during curing [33]. This increase may be interpreted as an effect of the sealing action of graphene oxide, which stabilizes moisture retention in specimens stored under varying environmental conditions.

Overall, the changes in porosity of the modified material lead to the closure of the pore system involved in fluid transport. This effect intensifies with curing time and with the development of a microstructure containing the smallest pores, characterized by a high specific surface area available for sorption. Consequently, this enables the stabilization of phases involved in the mechanisms of autogenous healing of the investigated set cement specimens.

## 4. Conclusions

1. GO-modified cement slurries have potential to extend the service life of well infrastructure by improving well integrity through enhancing microstructural properties, self-healing capacity, and resistance of the resulting set cement to environmental factors.
2. The experimental investigations demonstrated that the incorporation of graphene oxide significantly modifies the microstructure of the cement matrix in nine-year-old set cement and supports self-healing mechanisms. The long-term preservation of self-healing capacity is associated with the stable presence of reactive portlandite in the GO-modified cement matrix and the ability of this portlandite to migrate into open spaces, such as newly formed microcracks.
3. These results confirm that the addition of GO may promote densification of the set cement microstructure and limit the transport of corrosive agents (e.g., CO_2_) into the cement matrix under long-term curing conditions.
4. Modification of the cement slurry with GO limits carbonation penetration into the set cement over the long term. In nine-year-old specimens containing GO, calcite was detected mainly in the near-surface layer, whereas portlandite dominated in the deeper zones. In the reference specimen without GO, calcite was detected throughout the entire analyzed depth range, indicating deeper carbonation.
5. Long-term curing of GO-modified cement slurries leads to reorganization of the pore structure of the resulting set cement, characterized by a reduction in capillary porosity involved in fluid transport and an increase in the proportion of finer pores. These changes are accompanied by an increase in specific surface area, indicating reorganization of the set cement microstructure.

## Figures and Tables

**Table 1:** Selected physicochemical properties of CEM I 42.5 R cement [24].

Properties	Unit	Mean Value
Specific surface area (Blaine)	[cm^2^/g]	3800
Initial setting time	[min]	205
Soundness	[mm]	0.5
Loss on ignition	[%]	3.11
Insoluble residue	[%]	0.69
SO_3_	[%]	2.81
Cl^−^	[%]	0.08
Na_2_O_eq_	[%]	0.70

**Table 2:** Summary of selected porosity parameters (desorption).

Sample	BET Surface Area	Single Point Pore < 0.94 nm Volume	BJH Pore 1.7–300.0 nm Volume	BET Average Pore Diameter (4V/A)	BJH Average Pore Width (4V/A)
	m^2^/g	cm^3^/g	cm^3^/g	nm	nm
S0.7	17.7811	0.0902	0.0899	20.31	15.30
S0.7_0.15	21.5093	0.1072	0.1069	19.93	14.71
N0.7	19.5376	0.0038	0.0592	0.78	9.60
N0.7_0.05	75.3828	0.0183	0.1887	0.96	8.42
N0.7_0.15	83.2733	0.0202	0.1845	0.97	7.64

## Data Availability

The data presented in this study are available on request from the corresponding author.
